# CD244 overexpression indicates NK cell dysfunction and tumor progression in diffuse large B-cell lymphoma

**DOI:** 10.3389/fimmu.2026.1855521

**Published:** 2026-07-07

**Authors:** Yiming Yang, Biyang Zeng, Huimin Yang, Xin Liu, Peilin Li, Chao Mai, Shunhai Jian, Qiqi Zhu

**Affiliations:** 1 Department of Pathology, Affiliated Hospital of North Sichuan Medical College, Nanchong, China; 2 Institute of Basic Medicine, North Sichuan Medical College, Nanchong, China; 3 Department of Emergency, Affiliated Hospital of North Sichuan Medical College, Nanchong, China

**Keywords:** CD244, clinicopathological analysis, diffuse large B-cell lymphoma, immunotherapy, natural killer cells, prognosis, single-cell RNA sequencing, tumor microenvironment

## Abstract

**Background:**

CD244, expressed in the tumor microenvironment (TME), is associated with impaired function of natural killer (NK) and T cells; however, its role in diffuse large B-cell lymphoma (DLBCL) remains poorly understood. This study aimed to elucidate the immunological significance and regulatory mechanisms of CD244 in DLBCL.

**Methods:**

Using single-cell and bulk RNA sequencing, we analyzed CD244 expression patterns, its major intracellular adaptor molecules, and correlations with immune checkpoints, metabolic alterations, and immune activity. The clinical and biological implications of CD244 expression, including associations with clinicopathological characteristics, TME composition, prognosis, and response to immune checkpoint blockade (ICB) therapy, were investigated by integrating single-cell and bulk RNA sequencing, immunohistochemistry, and reverse transcription-quantitative polymerase chain reaction. Intercellular communication networks, key transcription factors, and somatic mutations were further evaluated to uncover regulatory mechanisms.

**Results:**

NK cells were the primary CD244-expressing population in DLBCL, co-overexpressing PDCD1, CTLA4, LAG3, TIGIT, PTGER4, and CD160. EAT2 was identified as CD244’s predominant intracellular adaptor, suggesting that CD244-associated inhibitory signaling may contribute to metabolic dysregulation and immune dysfunction in NK cells. Elevated CD244 expression correlated with an immunosuppressive TME, worse clinicopathological features, poorer outcomes, and increased potential responsiveness to ICB. Furthermore, CD244 expression may be regulated by STAT3 activation in NK cells and ASXL3 mutations in tumor cells via the BTLA–TNFRSF14 pathway.

**Conclusions:**

This study highlights the critical role of CD244 in NK cell dysfunction and DLBCL progression, providing a promising target for optimizing immunotherapy.

## Highlights

- What is already known on this topic – CD244 is implicated in the dysfunction of natural killer (NK) cells and T cells. It exhibits great therapeutic potential, while little is known about diffuse large B-cell lymphoma (DLBCL).
- What this study adds – CD244 may contribute to the exhaustion and immune deficiency of NK cells and is correlated with immunosuppressive tumor microenvironment, severe clinicopathological features, adverse clinical outcomes, and increased susceptibility to immune checkpoint blockade therapy.
- How this study might affect research, practice or policy – Our study clarified the significance of CD244 in NK cells dysfunction and DLBCL progression, which offered a feasible option for optimizing immunotherapy.

## 1 Introduction

Diffuse large B-cell lymphoma (DLBCL) constitutes around 40% of non-Hodgkin lymphoma, demonstrating extensive heterogeneity in phenotype, molecular characteristics, and clinical features (1). The standard therapy based on rituximab, cyclophosphamide, doxorubicin, vincristine, and prednisone (R-CHOP) has shown favorable therapeutic effects in DLBCL (2). At the same time, about 40% of patients are resistant to front-line treatment or suffer from relapsed disease (3). Therefore, effectively combined therapeutics remain an unmet need to improve the prognosis of DLBCL patients.

The disturbance of the tumor microenvironment (TME) of DLBCL, mainly composed of tumor-infiltrating lymphocytes (TILs), natural killer (NK) cells, macrophages, and dendritic cells (DC), plays a critical role in DLBCL initiation, progression, and therapeutic resistance (4, 5). Immunotherapy targeting TME-related factors, such as immune checkpoint blockade (ICB) therapy (6), has revolutionized the treatment of many solid tumors and lymphohematopoietic malignancies, such as lung cancer (7), breast cancer (8), melanoma (9), acute myeloid leukemia (10), and Hodgkin lymphoma (11), but shown limited benefits in DLBCL (12). Meanwhile, durable responses are only achieved in a small subset of patients, and novel immune targets are urgently required to prolong the survival of patients. Recent work has shown that targeting inhibitory immunomodulatory molecules such as CD5 can enhance persistence, reduce exhaustion, and improve antitumor efficacy of engineered T cells in hematologic malignancies, further underscoring the translational importance of identifying additional immunoregulators in lymphoma (13, 14).

CD244, a member of the signaling lymphocyte-activation molecule (SLAM) family, is expressed on many immune cell types, predominantly including NK cells, CD8^+^TILs, DC, macrophages, and myeloid-derived suppressor cells (15). CD244 is critically engaged in the cytotoxic function and effector molecules production of NK cells and CD8^+^TILs and plays a pivotal role in regulating immune responses (16). Interestingly, recent studies suggested the double-edged sword role of CD244 in the function modulation of immune cells and cancer progression (17). CD244 could elicit stimulatory or inhibitory effects upon the interaction with its ligand CD48, depending on the context (15), and the inhibitory signals are implicated in the impaired immune function of NK cells (18), CD8^+^TILs (15), DC (16), and macrophages (19). CD244 has emerged as a novel inhibitory immune checkpoint, prognosticator, and potential therapeutic target in many types of cancers (20). However, the exact role of CD244 in DLBCL onset and progression remains elusive.

In this study, the expression of CD244 was compared between DLBCL and control samples. The primary cell types that expressed CD244, the expression of its intracellular adaptor molecules, and the relationships between CD244 expression, canonical IC, metabolic changes, and immunological function in DLBCL were all thoroughly examined. The significance of CD244 expression in clinicopathological features, TME composition, prognosis evaluation, and ICB therapy efficacy was investigated. Moreover, the intercellular communications, the crucial transcription factors, and the potential somatic mutation were evaluated to reveal the possible regulatory mechanism of CD244 expression in DLBCL. This study aims to clarify the significance of the CD244 expression in DLBCL, which may facilitate the development of individualized immunotherapy.

## 2 Materials and methods

### 2.1 Patients

The Department of Pathology at the Affiliated Hospital of North Sichuan Medical College identified 153 DLBCL cases diagnosed prior to chemotherapy between January 2016 and March 2025. The diagnoses were given in accordance with the DLBCL diagnostic criteria specified by the World Health Organization (4th edition, 2018). Individuals with a history of secondary DLBCL and recurrent DLBCL were not included. Electronic medical records and pathological data analysis were used to obtain relevant clinicopathological information. In August 2025, survival data was gathered by computerized medical record reviews or telephone interviews. From the date of pathology diagnosis to the date of death or the most recent follow-up, the overall survival (OS) time was computed.

### 2.2 Single-cell RNA sequencing

The Gene Expression Omnibus (GEO) database (GSE182434 and GSE182436) (21) and the heiDATA database (22) provided the genomics data of single-cell RNA sequencing (scRNA-seq) of DLBCL (n=7) and healthy controls (n=4), including three reactive lymph nodes and one tonsil tissue sample. The scRNA-seq data analysis was carried out in accordance with our earlier research (23, 24). Single-cell metabolism in NK cells from DLBCL and healthy controls was measured using the ScMetabolism program. The GSEA approach was used to determine the immune-related pathway activity in NK cells. To compute cell-cell interactions centered on NK cells in DLBCL, the CellChat program was utilized.

### 2.3 Gene expression profiling data

The GEO database’s GSE181063 (n=1310), GSE117556 (n=928), GSE31312 (n=470), GSE10846 (n=412), GSE32918 (n=249), GSE87371 (n=221), GSE11318 (n=200), and GSE53786 (n=119) provided the gene expression profiling data and pertinent clinicopathological data for DLBCL patients. All eight cohorts are microarray-based datasets. Processed series matrix files were downloaded from GEO, log2-transformed, and quantile-normalized. These data were analyzed in accordance with our earlier research (23, 24). Immune cell infiltration in DLBCL was examined using CIBERSORT (https://cibersort.stanford.edu/). The response to ICB therapy in DLBCL was forecast using the Tumor Immune Dysfunction and Exclusion (TIDE) score (http://tide.dfci.harvard.edu/).

### 2.4 Immunohistochemistry

CD244 protein expression was examined using immunohistochemistry (IHC) on formalin-fixed, paraffin-embedded (FFPE) lymph node tissue slices of DLBCL (n=153). Anti-CD244 (Proteintech, Catalog: 16677-1-AP) was the primary antibody for CD244. CD244 expression was mainly detected in the cytoplasm or cell membrane of TILs from DLBCL. The mean numbers of CD244^+^ cells per high-power field (HPF) at the hot spot area were used to manually assess the stains.

### 2.5 Reverse transcription-quantitative polymerase chain reaction

Following the manufacturer’s instructions, the RNeasy FFPE Kit (QIAGEN, 73504) was used to extract the total RNA from FFPE lymph node tissue slices of DLBCL (n=95). The following primers were used for CD244 and ACTIN in reverse transcription-quantitative polymerase chain reaction (RT-qPCR): CD244 forward, GGGAGAATGGCTCTTTGCCT; CD244 reverse, GCTGAGCTGCCTTGATGAGA; ACTIN forward, CCGCGAGAAGATGACCCAGA; ACTIN reverse, GATAGCACAGCCTGGATAGCA. A Bio-Rad CFX manager was used to quantify the samples, and ACTIN was assigned as the reference gene.

### 2.6 Somatic mutation analysis

The TCGAbiolinks software was used to retrieve the somatic mutation profile of DLBCL (n=48) from the Cancer Genome Atlas (TCGA) database. Sangerbox (http://sangerbox.com/) was used to globally compare mutation frequencies of frequently mutated genes between CD244- high and CD244- low groups. Genes showing differential mutation patterns were then further examined.

### 2.7 Statistical analyses

Statistical analyses were conducted using R software (version 4.2.1) and the Statistical Package for the Social Sciences (SPSS) version 26.0 software (SPSS Corp., Chicago, IL, USA). Numerical data was evaluated using the nonparametric test, while categorical data was compared using the Chi-squared test and the Mann-Whitney U-test. The relationship between CD244 expression and NKG7, GNLY, PDCD1, CTLA4, HAVCR2, LAG3, TIGIT, PTGER4, CD160, and EAT2/SH2D1B was examined using Spearman’s correlation coefficients. The cut-off value for CD244 in OS prediction is estimated using receiver operating characteristic curves. The OS time was computed using the Kaplan-Meier estimate. A two-tailed p-value of less than 0.05 was deemed statistically significant.

## 3 Results

### 3.1 The expression pattern of CD244 in DLBCL

**Figure 1:**
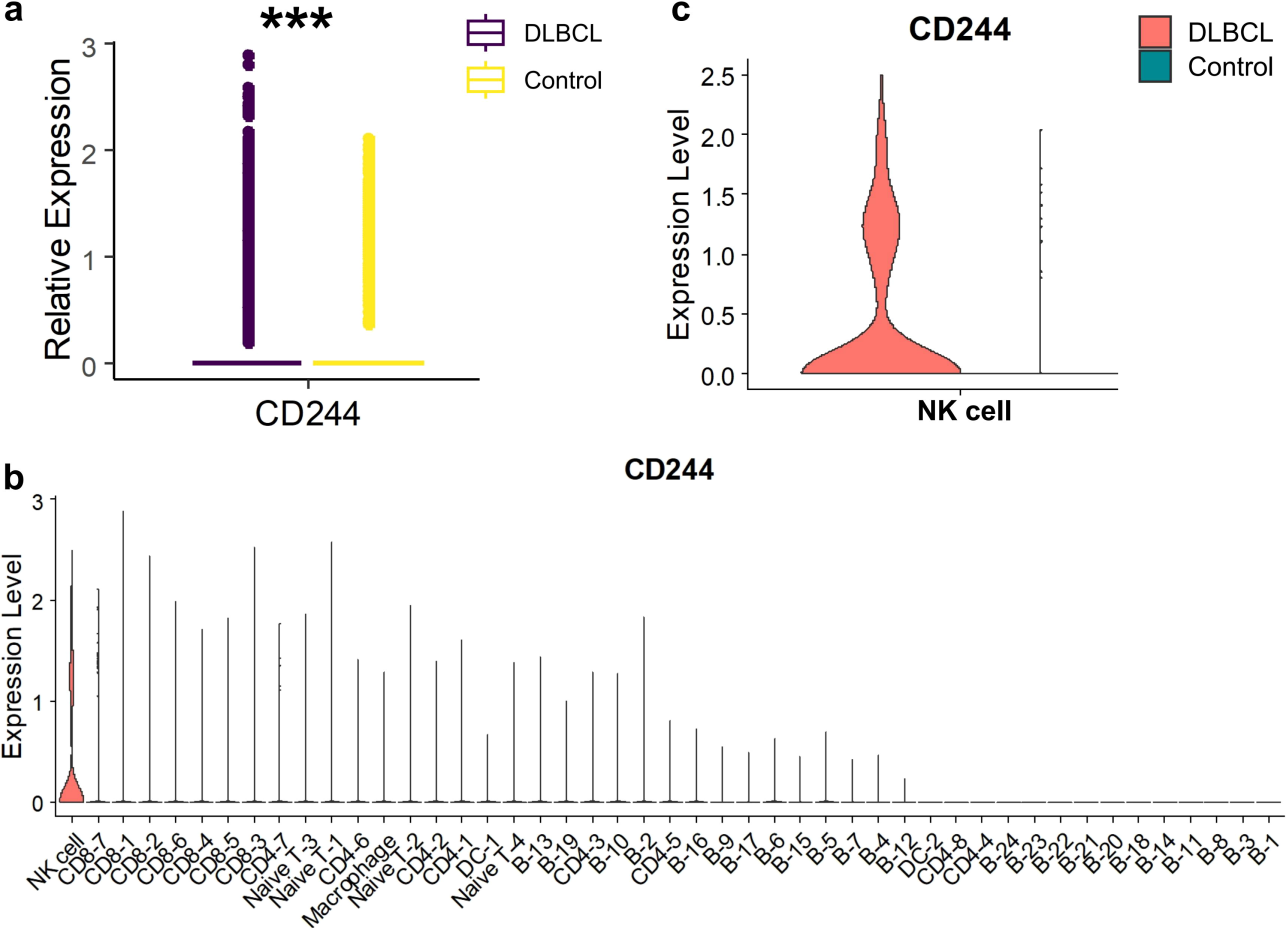
The expression pattern of CD244 in DLBCL detected by scRNA-seq. **(a)** The expression of CD244 between DLBCL and healthy controls; **(b)** The expression of CD244 in 47 cell clusters from DLBCL; **(c)** The expression of CD244 in NK cells from DLBCL and healthy controls. ***: p<0.001.

ScRNA-seq was used to obtain single-cell transcriptomes of DLBCL (n=7) and healthy controls (n=4). The results revealed that CD244 expression was considerably higher in DLBCL than in healthy controls (**Figure 1a**) (p<0.001). Using UMAP analysis, all cells were further divided into 47 subsets according to the corresponding marker genes (**Supplementary Figure 1**). The outcome demonstrated that CD244 was highly expressed in NK cells (**Figure 1b**).

The expression of marker genes (NKG7, GNLY) was used to determine single-cell gene expression in NK cells from DLBCL (n=208) and healthy controls (n=72) for further examination. NK cells from DLBCL showed increased expression of CD244 when compared to healthy controls (**Figure 1c**).

### 3.2 The correlation between CD244 and IC expression in DLBCL

**Figure 2:**
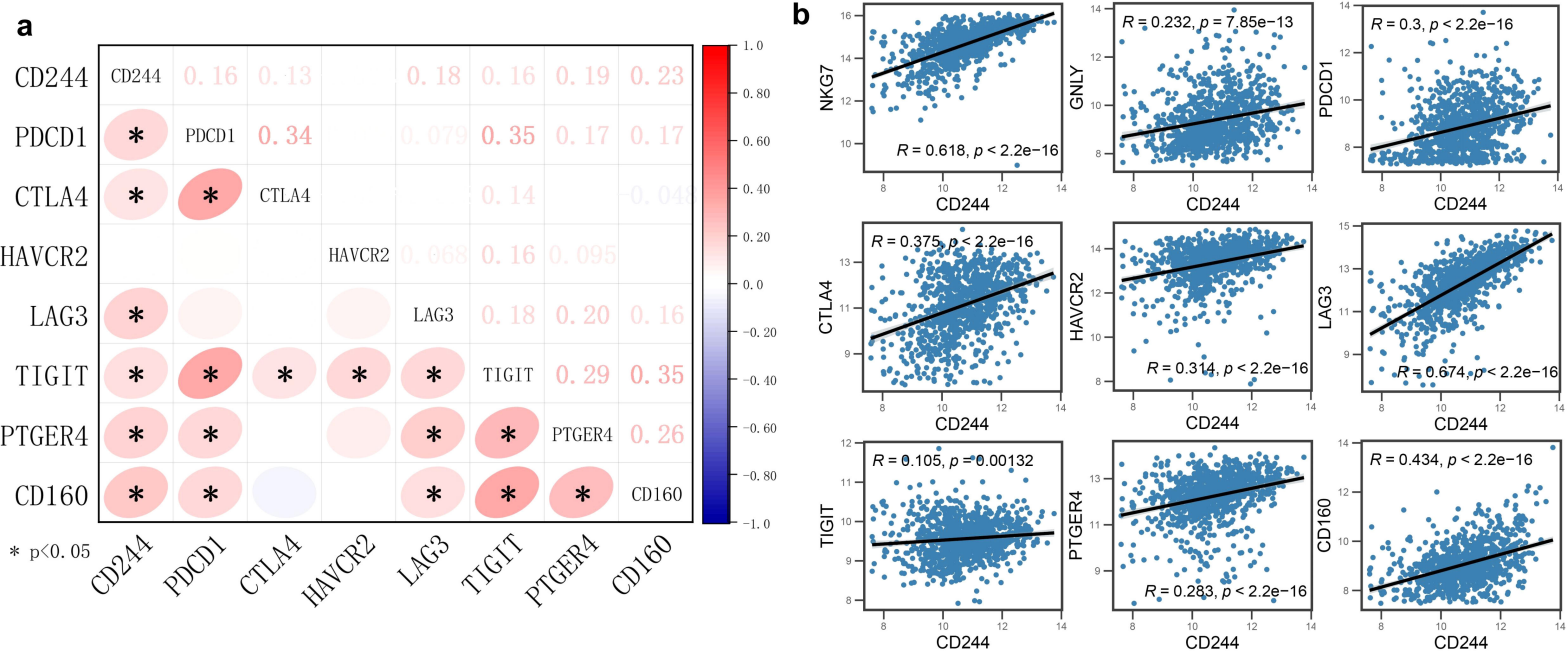
The correlation between CD244 and IC expression in DLBCL. **(a)** The correlations among CD244, PDCD1, CTLA4, HAVCR2, LAG3, TIGIT, PTGER4, and CD160 in NK cells detected by scRNA-seq; **(b)** The correlations among CD244, NKG7, GNLY, PDCD1, CTLA4, HAVCR2, LAG3, TIGIT, PTGER4, and CD160 in DLBCL detected by RNA sequencing. *: p<0.05.

The correlation between CD244 and IC expression in NK cells was analyzed by scRNA-seq, and the result showed that CD244 was positively correlated with PDCD1 (r=0.16, p=0.007), CTLA4 (r=0.13, p=0.031), LAG3 (r=0.18, p=0.002), TIGIT (r=0.16, p=0.007), PTGER4 (r=0.19, p=0.001), and CD160 (r=0.23, p<0.001) (**Figure 2a**). No significant correlation was observed between CD244 and HAVCR2 (r =-0.0075, p = 0.9). The correlations among CD244, NK cells infiltration, and IC expression were further analyzed based on RNA sequencing data of DLBCL, and the result showed that CD244 was positively correlated with NKG7 (r=0.618, p<0.001), GNLY (r=0.232, p<0.001), PDCD1 (r=0.3, p<0.001), CTLA4 (r=0.375, p<0.001), HAVCR2 (r=0.314, p<0.001), LAG3 (r=0.674, p<0.001), TIGIT (r=0.105, p=0.001), PTGER4 (r=0.283, p<0.001), and CD160 (r=0.434, p<0.001) (**Figure 2b**).

### 3.3 The main intracellular adaptor molecules of CD244 in DLBCL

**Figure 3:**
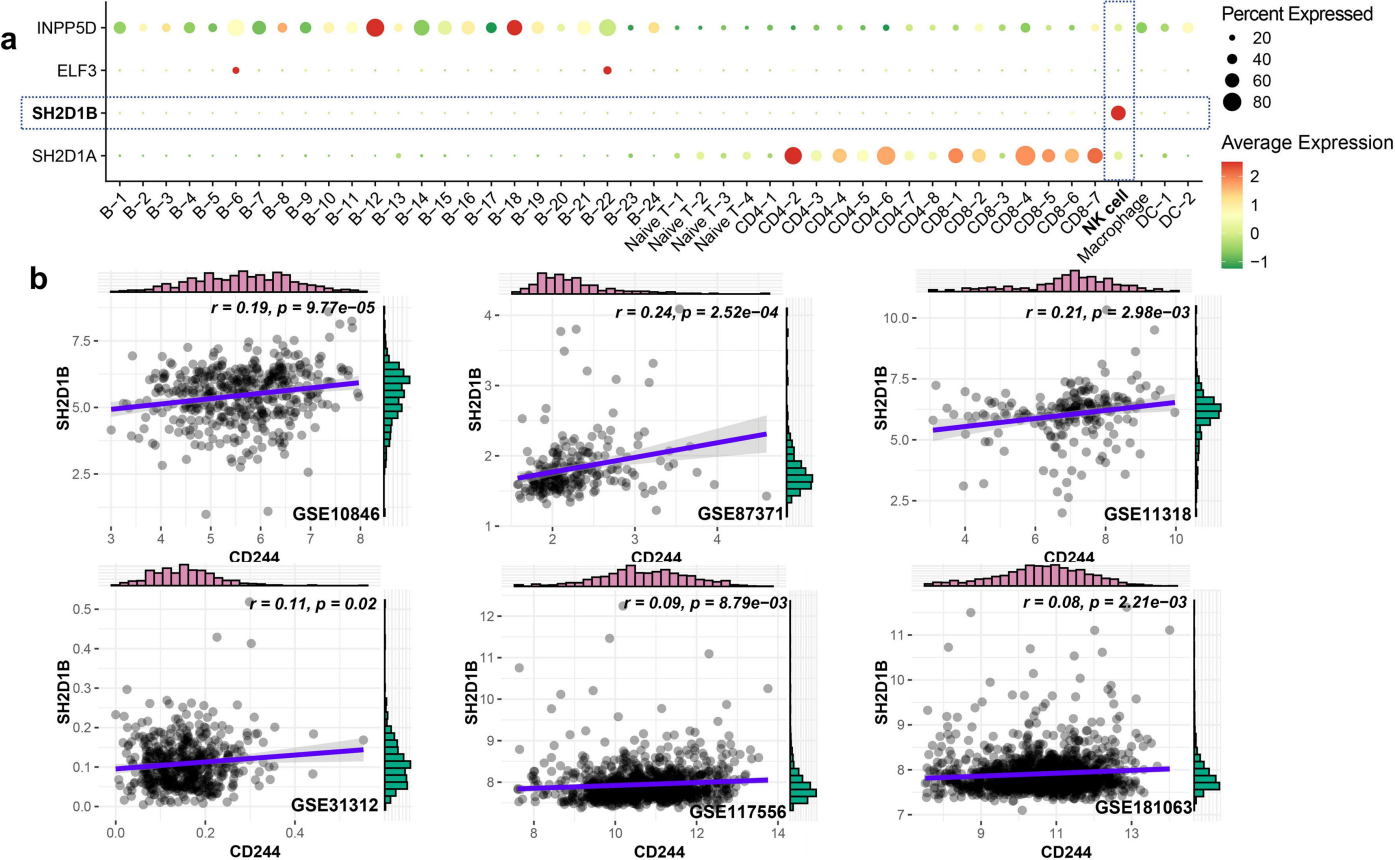
The main intracellular adaptor molecules of CD244 in DLBCL. **(a)** The expression of SAP/SH2D1A, EAT2/SH2D1B, SHIP/INPP5D, and ERT/ELF3 in 47 cell clusters from DLBCL detected by scRNA-seq; **(b)** The correlation between CD244 and EAT2/SH2D1B in DLBCL detected by RNA sequencing.

The expression of major intracellular adaptor molecules of CD244 in NK cells, including SAP/SH2D1A, EAT2/SH2D1B, SHIP/INPP5D, and ERT/ELF3, was analyzed by scRNA-seq (**Figure 3a**). The result showed that expression of SAP/SH2D1A and SHIP/INPP5D were detected in various clusters in DLBCL, represented by CD4^+^TILs and CD8^+^TILs, while their expression was relatively low in NK cells, and ERT/ELF3 was expressed in B cells. Meanwhile, EAT2/SH2D1B was mainly expressed in NK cells. Further analysis by RNA sequencing data of DLBCL consistently identified the significant positive correlation between CD244 and EAT2/SH2D1B expression on the histology level (**Figure 3b**).

### 3.4 The significance of CD244 in the metabolism and immune activity of NK cells

**Figure 4:**
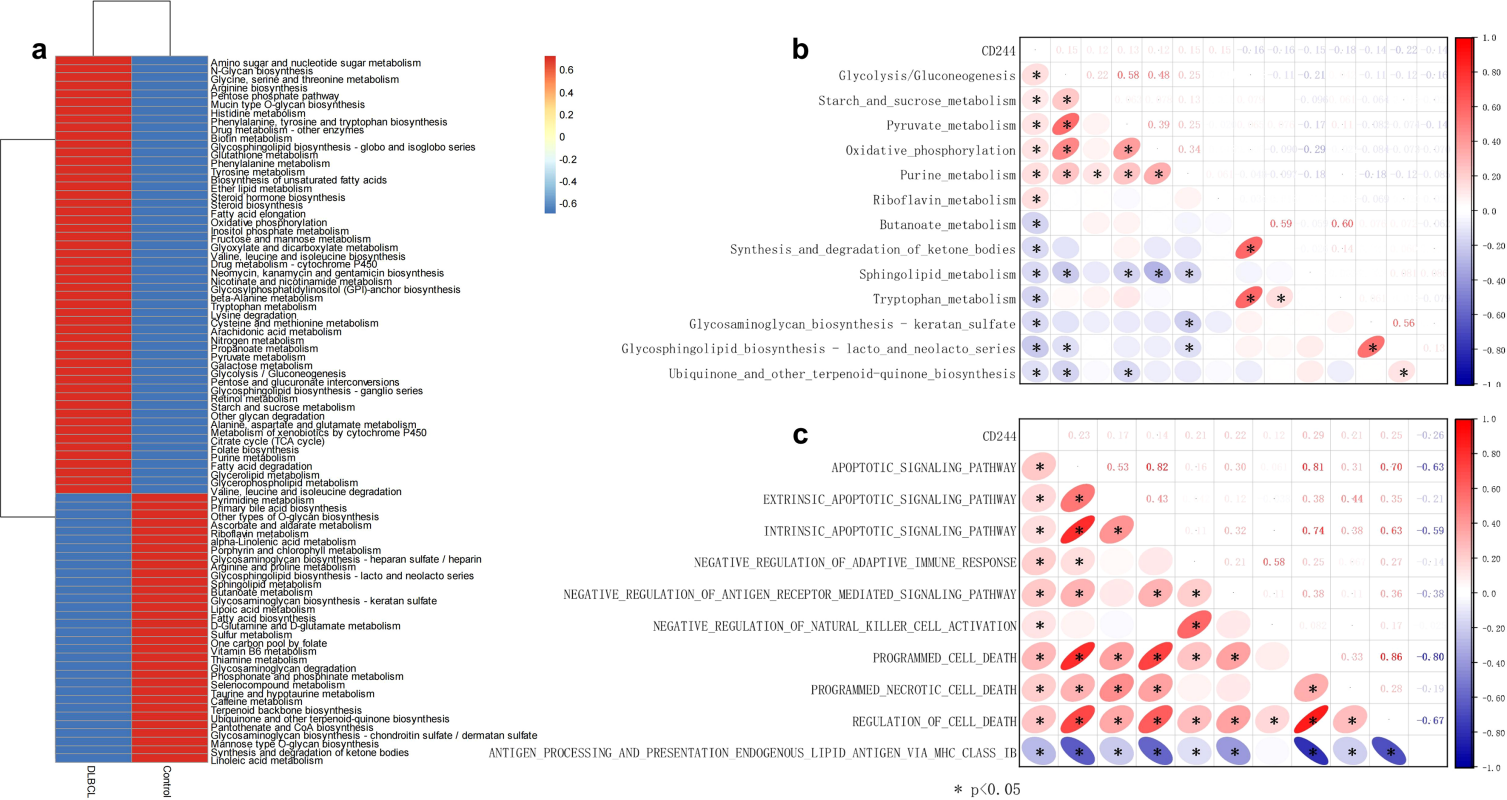
The significance of CD244 in the metabolism and immune activity of NK cells detected by scRNA-seq. **(a)** The metabolic alterations in NK cells between DLBCL and healthy controls; **(b)** The correlation between CD244 expression and metabolic alterations in NK cells from DLBCL; **(c)** The correlation between CD244 expression and immune-related activities in NK cells from DLBCL. *: p<0.05.

The metabolic status in NK cells was evaluated by scMetabolism, and the result showed a marked difference between DLBCL and healthy controls (**Figure 4a**). Further analysis showed that CD244 expression was positively correlated with Glycolysis/Gluconeogenesis, Oxidative phosphorylation, Purine metabolism, Pyruvate metabolism, and Starch and sucrose metabolism, while negatively correlated with Butanoate metabolism, Sphingolipid metabolism, and Tryptophan metabolism (**Figure 4b**).

The immune-related activities in NK cells were evaluated by GSEA, and the result showed that CD244 expression was positively correlated with Apoptotic signaling pathway, Negative regulation of adaptive immune response, Negative regulation of Natural killer cell activation, and Programmed cell death, while negatively correlated with Antigen processing and presentation endogenous lipid antigen via MHC class Ib (**Figure 4c**).

### 3.5 The correlation between CD244 and clinicopathological features

**Figure 5:**
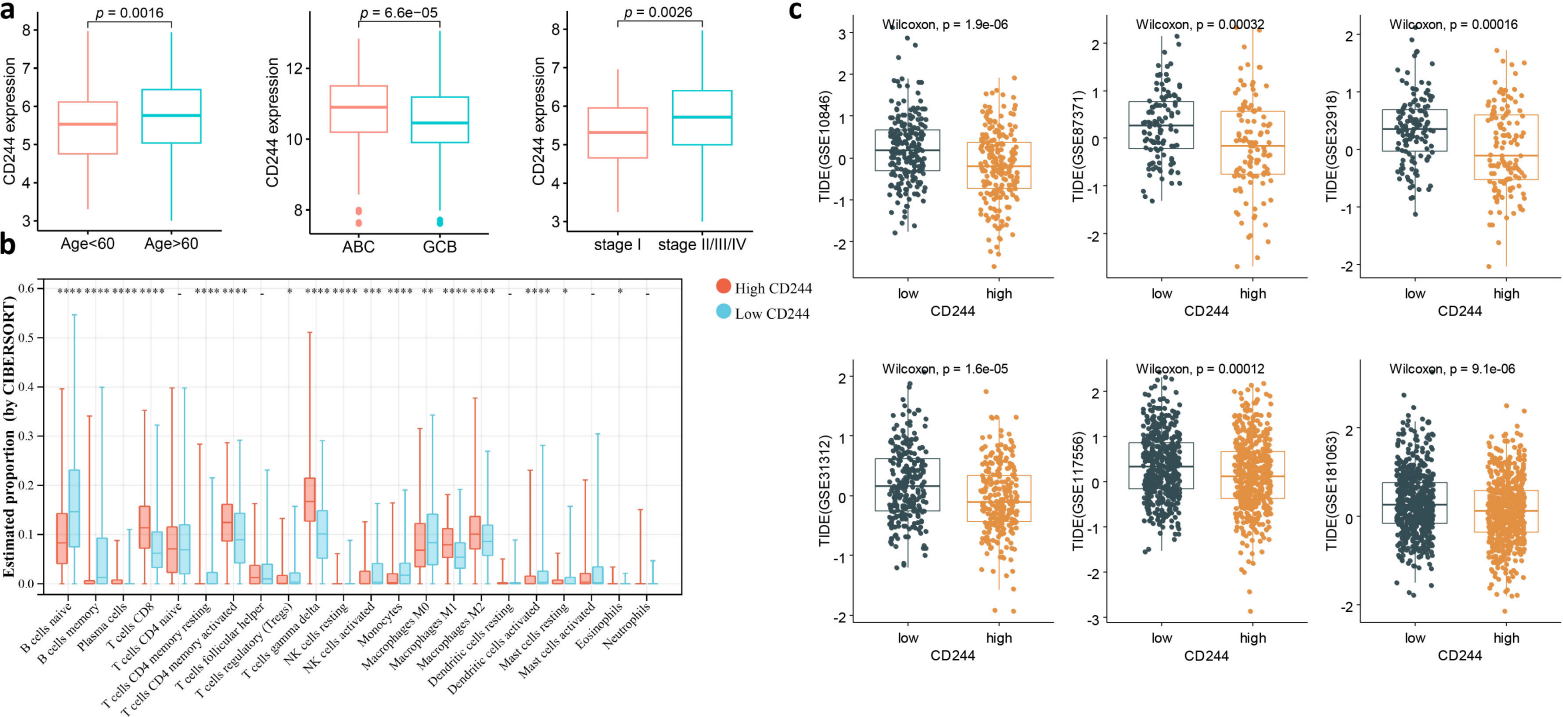
The significance of CD244 expression in DLBCL detected by RNA sequencing. **(a)** The correlation between CD244 and clinicopathological features; **(b)** The significance of CD244 in TME composition; **(c)** The correlation between CD244 expression and TIDE scores across six independent DLBCL cohorts. Lower TIDE scores predict a potentially higher likelihood of ICB responsiveness. *: p<0.05, **: p<0.01, ***: p<0.001, ****: p<0.0001.

The correlation between CD244 expression and clinicopathological features is shown in **Figure 5a**. Compared with the low CD244 group, patients within the high CD244 group were presented with a higher proportion of old age (>60 years old) (p=0.001), activated B-cell-like (ABC) subtype (p<0.001), and advanced stage (III/IV) (p=0.002).

### 3.6 The significance of CD244 in TME composition

The composition of TME in DLBCL was analyzed by CIBERSORT, and the correlation between CD244 expression and immune cell infiltration was assessed. The results showed that compared with the low CD244 group, a higher proportion of M2 macrophages, and a lower proportion of activated NK cells, activated DC, and memory B cells were identified in the high CD244 group (**Figure 5b**).

### 3.7 The significance of CD244 in ICB therapy

TIDE scores were assessed across six independent DLBCL cohorts (GSE181063, GSE117556, GSE31312, GSE10846, GSE32918, GSE87371). Compared with the CD244- low group, the CD244- high group consistently exhibited lower TIDE scores (**Figure 5c**), predicting a potentially higher likelihood of ICB responsiveness.

### 3.8 The significance of CD244 in prognosis evaluation

**Figure 6:**
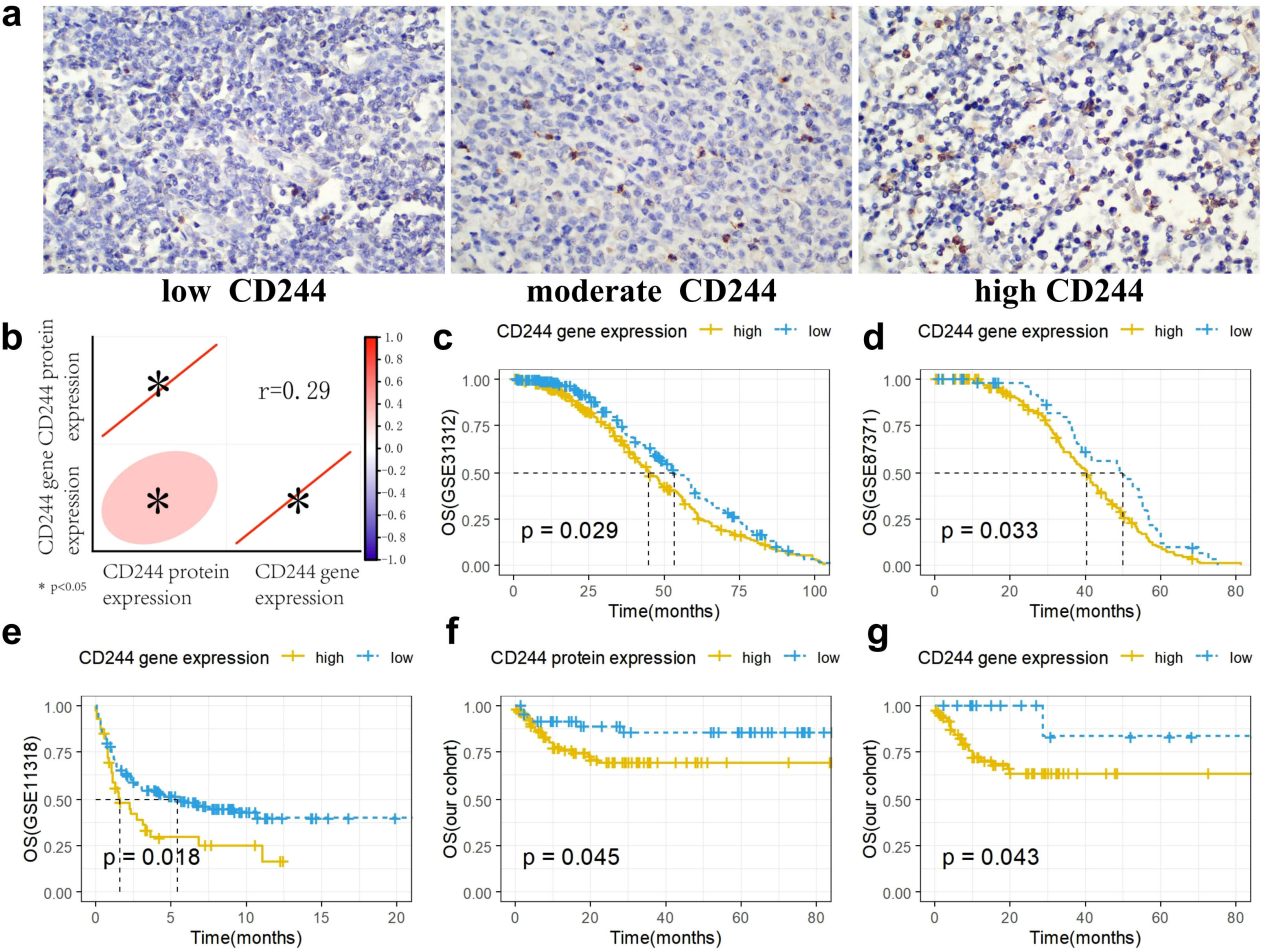
The significance of CD244 in prognosis evaluation. **(a)** Representative IHC staining of CD244 protein in DLBCL tissues. **(b)** Spearman correlation between CD244 protein expression (IHC) and gene expression (RT-qPCR) in DLBCL (n = 95). **(c)** Kaplan-Meier estimates of overall survival according to CD244 expression in GSE31312 (n = 470). **(d)** Kaplan-Meier estimates of overall survival according to CD244 expression in GSE87371 (n = 221). **(e)** Kaplan-Meier estimates of overall survival according to CD244 expression in GSE11318 (n = 200). **(f)** Kaplan-Meier estimates of overall survival according to CD244 protein expression (IHC) in our cohort (n = 153). **(g)** Kaplan-Meier estimates of overall survival according to CD244 mRNA expression (RT-qPCR) in our cohort (n = 95). Log rank test was used for all survival comparisons. *: p<0.05.

IHC and RT−qPCR were used to further examine the protein and gene expression of CD244 in DLBCL from our cohort of 153 patients. The median follow−up was 19.3 months (range: 0.1–87 months), and 30 death events were recorded. **Table 1** summarizes the basic data of patients with DLBCL. IHC revealed that the mean number of CD244^+^ cells (0–40/HPF) was 3/HPF (**Figure 6a**). The CD244 protein and gene expression were found to positively correlate (p<0.05) (**Figure 6b**). The DLBCL patients were then divided into high and low groups based on the protein or gene expression of CD244. Based on the GEO datasets, the association between CD244 expression and DLBCL patients’ survival was assessed and confirmed by our cohort. Patients with high CD244 expression showed shorter OS than those with low CD244 expression in GSE31312 (n=470) (p=0.029) (**Figure 6c**), GSE87371 (n=221) (p=0.033) (**Figure 6d**), GSE11318 (n=200) (p=0.018) (**Figure 6e**), and our cohort (IHC, n=153; RT-qPCR, n=95) (p<0.05) (**Figures 6f, g**). In multivariable analysis of our cohort (**Table 2**), patients with high CD244 expression also tend to demonstrate an increased risk of death (p = 0.148).

**Table 1:** Baseline characteristics of patients with DLBCL.

Characteristic	No	in group(%)
Age, mean (range)	61.5(25-88)	
Age		
>60 years	88/153	57.5
≤60 years	65/153	42.5
Gender		
Male	83/153	54.2
Female	70/153	45.8
COO		
GCB	42/151	27.8
Non-GCB	109/151	72.2
PS		
0-1	107/147	72.8
2-5	40/147	27.2
Stage		
I/II	59/148	39.9
III/IV	89/148	60.1
IPI		
0-1	58/148	39.2
2-5	90/148	60.8
B-symptom		
yes	48/143	33.6
no	95/143	66.4
Primary site		
Nodal	82/149	55
Extranodal	67/149	45
LDH>220 IU/L		
yes	53/106	50
no	53/106	50
Treatment		
CHOP- based therapy	115/132	87.1
Other therapy	17/132	12.9
Response to CHOP		
CR+PR	64/95	67.4
SD+PD	31/95	32.6

COO, cell of origin; GCB, germinal center B-cell-like; PS, performance status; LDH, lactate dehydrogenase; CHOP, cyclophosphamide, doxorubicin, vincristine, prednisone; CR, complete remission; PR, partial remission; SD, stable disease; PD, progressive disease.

**Table 2:** Multivariate Cox regression analyses with relative risk of overall survival (death due to any cause) estimated as hazard ratios with 95% confidence intervals and p values by putative prognostic factors in patients with DLBCL.

		Multivariate	
Clinicopathological characteristics	Group	HR(95%)	*p*
CD244 protein expression	high vs low	1.939:0.774-4.857	0.148
Age	≥60 vs <60	3.940:1.134-13.765	0.031
Gender	yes vs no	0.915:0.291-2.872	0.566
Stage	III/IV vs I/II	1.835:0.393-8.557	0.188
IPI	2-5 vs 0-1	1.880:0.593-5.960	0.283
PS	2-5 vs 0-1	3.268:1.242-18.599	0.016
B symptoms	yes vs no	1.588:0.567-4.446	0.228
Primary site	Nodal vs Extranodal	1.049:0.353-3.119	0.993
LDH>220 IU/L	yes vs no	2.444:0.682-8.759	0.133

### 3.9 The possible regulatory mechanism of CD244 in DLBCL

**Figure 7:**
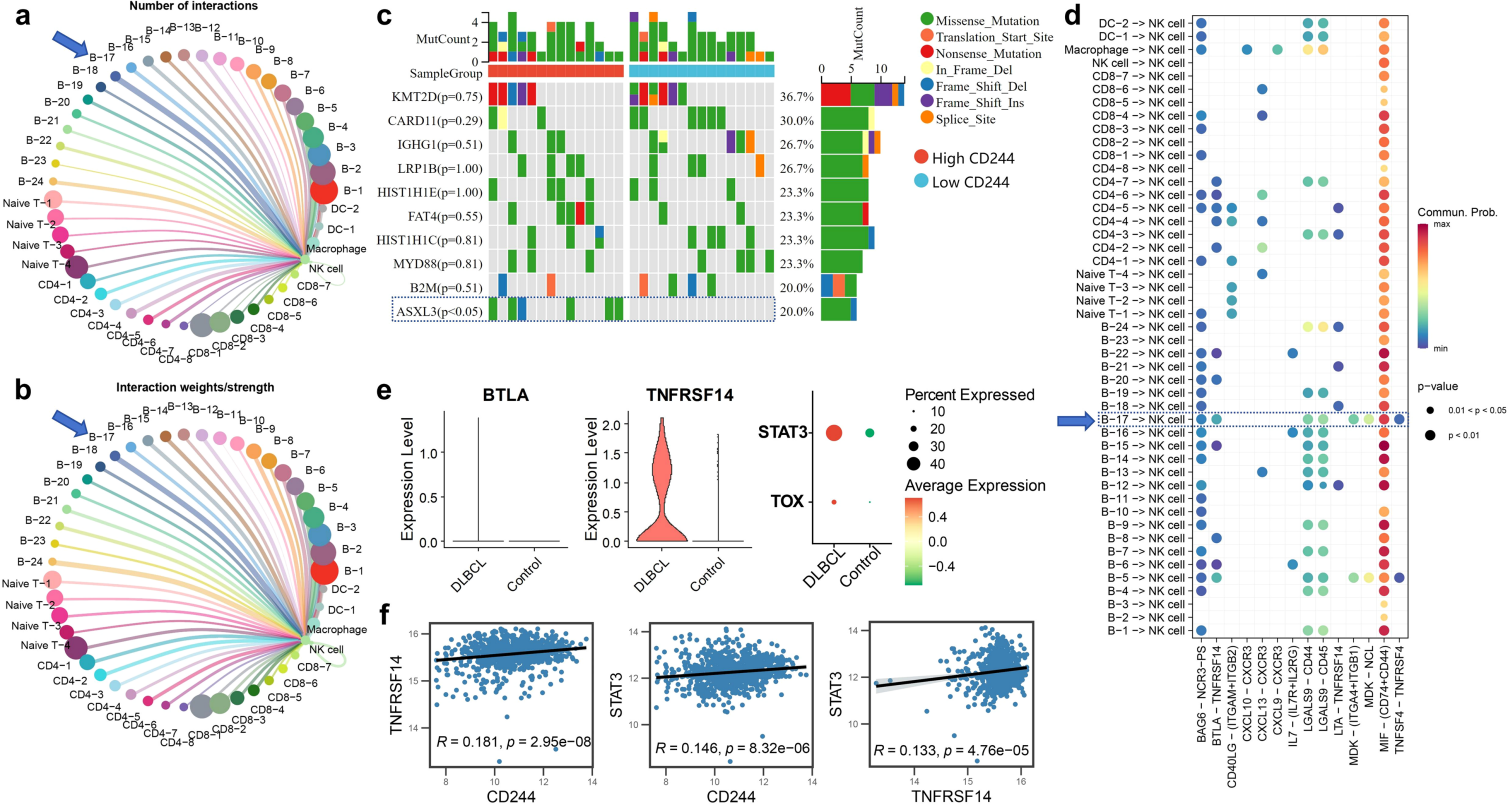
The possible regulatory mechanism of CD244 expression in DLBCL. **(a, b)** The interaction numbers **(a)** and weights/strength **(b)** between NK cells and other components in DLBCL detected by scRNA-seq. In both panels, node size represents the number of cells in each cluster. Edge thickness indicates the number of inferred ligand–receptor pairs in **(a)** and communication probability in **(b)**. All inferred interactions are displayed without additional filtering, and edge weights are globally scaled to the maximum value for visualization. Analysis was performed using CellChat with default parameters; **(c)** Genomic landscape of frequent somatic mutation in high and low CD244 expression group in DLBCL; **(d)** The interaction pathways between NK cells and other components in DLBCL detected by scRNA-seq; **(e)** The expression of BTLA, TNFRSF14, STAT3, and TOX in NK cells from DLBCL and healthy controls detected by scRNA-seq; **(f)** The correlations among CD244, TNFRSF14, and STAT3 in DLBCL detected by RNA sequencing.

The inter-cell communications between NK cells and other components in DLBCL were evaluated through scRNA-seq, and the results of interaction numbers and weights/strength both showed that the B-17 cluster tightly interacted with NK cells (**Figures 7a, b**). Meanwhile, copy number variation (CNV) analysis of B cells revealed the malignant property of the B-17 cluster (**Supplementary Figure 2**). Moreover, analysis of somatic mutation showed that, among the frequently mutated genes, only ASXL3 exhibited a significant difference between groups. ASXL3 mutations were detected in 6/15 (40%) of the CD244- high group versus 0/17 (0%) of the CD244- low group (Fisher’s exact test, p = 0.006; **Figure 7c**), suggesting the potential role of tumor cells in the regulation of CD244 in NK cells.

Further analysis demonstrated that multiple pathways were involved in the interactions between the B-17 cluster and NK cells, including BTLA-TNFRSF14, LGALS9-CD44, and CD74-CD44 (**Figure 7d**). The expression of BTLA and TNFRSF14 in NK cells was analyzed by scRNA-seq, and the result showed that TNFRSF14 was overexpressed in DLBCL, while BTLA was low in DLBCL and healthy controls (**Figure 7e**). The correlations among CD244, TNFRSF14, and key transcription factors (TFs) in NK cells were analyzed by scRNA-seq, and the results showed that STAT3 and TOX both positively correlated with CD244 (**Supplementary Table 1**) and TNFRSF14 expression (**Supplementary Table 2**). However, the overall expression of TOX was relatively low compared with STAT3 in NK cells (**Figure 7e**). Further analysis of RNA sequencing data confirmed the positive correlations among CD244, TNFRSF14, and STAT3 on the histology level (**Figure 7f**).

## 4 Discussion

Immunotherapy, including ICB treatment represented by PD-1 and CTLA4 blockade, has shown remarkable efficacy in many types of cancer over the past decade, while the related therapeutic value in DLBCL is limited, emphasizing the growing need for additional treatment approaches. CD244 is identified as a novel IC that transmits suppressive signals in immune response and could lead to the immune escape of tumor cells, and CD244 inhibition has great potential for inducing cancer regression and improving survival. However, the expression pattern and immune role of CD244, as well as the underlying regulatory mechanism in DLBCL, remain unknown, and further exploration may facilitate the optimization of therapeutic options.

Our study showed that the expression of CD244 is significantly higher in DLBCL compared with healthy controls, suggesting a potential role in cancer pathogenesis. Indeed, high CD244 expression in various immune cells, such as CD8^+^TILs, NK cells, and DC, could promote the formation of immunosuppressive TME and correlate with inferior prognosis in multiple types of cancer, including lung cancer (25), glioma (26), ovarian cancer (27), and esophageal cancer (28). Further analysis demonstrated that CD244 is predominantly expressed in NK cells from DLBCL, while rarely expressed in their counterparts from healthy controls. NK cells, crucial members of innate cytotoxic lymphocytes (29), are essential in the elimination of tumor cells, especially in lymphohematopoietic malignancies (30). Previous studies found the decrease of NK cells in DLBCL compared with healthy controls (31), and the proportion of NK cells from peripheral blood and TME both could affect the therapeutic efficacy (32), recurrence risk (33), and the outcome of patients with DLBCL (34–36). These highlighted the fundamental role of NK cells in anti-tumor immunity in DLBCL. However, recent studies have illustrated that NK cells exhibit an exhausted PD-1-enriched phenotype (37) and demonstrated compromised cytotoxic activity, degranulation, and inflammatory cytokine production in DLBCL (38). Based on the literature and our results, we speculated that high CD244 expression may be correlated with the impaired immune function of NK cells in DLBCL.

To elucidate the potential role of CD244 in NK cells, the correlations between CD244 and other IC expressions were evaluated in NK cells on the single-cell level and validated on the histology level. Our results showed that CD244 demonstrated significant positive correlations with canonical IC, including PD-1, CTLA4, LAG3, TIGIT, PTGER4, and CD160. Despite the positive role in immune response, high CD244 expression has also been linked to the dysfunction of T cells and NK cells within TME, particularly exhaustion phenotype with decreased cytokine production and increased apoptosis (18, 39–41). Meanwhile, NK cells with CD244 inhibition are endowed with increased cytotoxicity (42). The dual role of CD244 was mainly induced by its different intracellular adaptor molecules following the interaction with CD48 (17). SAP/SH2D1A, EAT2/SH2D1B, SHIP/INPP5D, and ERT/ELF3 are major intracellular adaptors for CD244 (15, 20, 43), and the CD244-SAP binding could potentiate the immune function of NK cells while CD244-EAT2 binding may be involved in the immunosuppressive signal (44, 45). Interestingly, our results showed that EAT2 is the dominant intracellular adaptor for CD244 in NK cells from DLBCL, and shows high specificity in NK cells compared with other components in TME. Combined with these findings, CD244 may mediate an inhibitor signal in NK cells through the binding to EAT2, accompanied by the over-expression of canonical IC, indicating the exhaustion and immune deficiency of NK cells in DLBCL.

Further analysis demonstrated that the metabolic status of NK cells from DLBCL is obviously different from that in healthy controls, and CD244 expression is closely correlated with the activities of multiple metabolic pathways in NK cells from DLBCL. Metabolic alterations play critical roles in T cell exhaustion, tumor immune evasion, and immunotherapy resistance (46, 47). Similar to solid tumors, metabolic alterations could also influence anti-tumor function, the responses to therapy, and prognoses in DLBCL (48–50). However, the metabolic alterations in NK cells from DLBCL have been seldom reported. Our results showed that high CD244 expression indicated high glycolysis/gluconeogenesis and oxidative phosphorylation activity in NK cells, both of which are implicated in exhausted immune microenvironment and treatment resistance in DLBCL (48). Meanwhile, our results suggested that high CD244 expression suggested increased activity of apoptosis and cell death-related pathways of NK cells, which was consistent with the previous study of hepatocellular carcinoma (18). In line with the exhaustion phenotype of NK cells, we further found that high CD244 expression indicated the impaired adaptive immune response, low activation activity, and the low antigen presentation ability of NK cells in DLBCL, implying the dysfunction of NK cells marked with over-expression of CD244. In brief, our study suggested the adverse influence of CD244 expression on the metabolic status and immune activity of NK cells, revealing the potential significance of CD244 in DLBCL progression.

The biological significance of CD244 expression in DLBCL was further investigated, and our results showed that the high CD244 expression group indicated an increase in M2 macrophages, and a decrease in activated NK cells, activated DC, and memory B cells. The immunosuppressive role of M2 macrophages in the TME that results in multiple pro-tumorigenic outcomes, including tumor progression, metastasis, dismal prognosis, and resistance to chemotherapy and ICB, has been well documented in malignant neoplasms, such as colorectal cancer (51), liver cancer (52), and mantle cell lymphoma (53). Interestingly, M2 macrophages could promote tumor growth by potentiating glycolysis activity, and inhibition of the related pathways led to effective tumor control and enhanced chemosensitivity in gastric cancer (54). Recently, M2 macrophages have been identified as key contributors to the exhaustion state and immune deficiency of CD8^+^TILs in DLBCL through the secretion of cholesterol (50). These studies revealed that M2 macrophages play critical roles in maintaining the immunosuppressive TME and may be involved in a metabolic manner in the dysfunction of NK cells in DLBCL patients with high CD244 expression. As expected, activated NK cells in the high CD244 group decreased, which may be the result of the inhibitor signal mediated by CD244, and was consistent with the low NK cell activation activity and compromised antigen presentation ability of NK cells in this study. Additionally, activated DC and memory B cells, key orchestrators in the adaptive immune response, immunosurveillance, and long-lived immunity (55, 56), both decreased along with the increase of CD244, possibly stressing the immune suppression correlated with high CD244. Taken together, the dysregulation of TME in the high CD244 group emphasized the potential suppressive role of CD244 in NK cells, which may collaborate with other components in TME to promote DLBCL progression. However, these TME compositions were inferred from gene expression data and warrant further validation at the protein level.

In addition, our results showed that high CD244 expression is of clinical significance in DLBCL, evidenced by a high proportion of patients with old age, ABC subtype, and advanced stage. These severe clinicopathological characteristics (57) may be the results of the CD244-associated immune deficiency of NK cells, which correlates with the poor prognosis of DLBCL patients.

The intercellular communications, TFs, and somatic mutation were further analyzed to explore the underlying regulatory mechanism of CD244 expression in NK cells. Our results showed that various cell types interacted with NK cells, among which B-17 exhibited the high CNV score (23), and was identified as the prominent subset in DLBCL. Our results indicated the complicated interactions between tumor cells and NK cells with high CD244, covering multiple pathways, such as BTLA, CXCL, GALECTIN, IL2, and MIF. The BTLA-TNFRSF14 dysregulation contributes to tumor escape and poor response to therapy in solid and lymphohematopoietic malignancies (58–60), and was involved in the NK cells immunosuppression and inferior prognosis (61). Our study demonstrated the increased expression of TNFRSF14 in NK cells from DLBCL compared with healthy controls, while BTLA is relatively lowly expressed in both cases. Meanwhile, analysis of the key TFs related to exhaustion showed significant positive correlations among STAT3, CD244, and TNFRSF14 in NK cells. STAT3 was linked to the exhaustion phenotypes of NK cells and CD8^+^TILs, and STAT3 suppression is beneficial for immune surveillance (23, 62). Besides, our results showed that the ASXL3 mutation was exclusively detected in the high CD244 group, implying the possible regulatory role of tumor cells in CD244 expression in DLBCL. Collectively, the CD244 expression may be regulated by STAT3 in NK cells and ASXL3 mutation in tumor cells through BTLA-TNFRSF14 pathway in DLBCL, which deserves further validation.

CD244 signaling disruption has shown potential therapeutic value through recovering the immune function of NK cells and T cells (39, 63). ICB therapy targeted at NK cell exhaustion has also enhanced the anti-tumor effect across many kinds of cancer (64–66), and CAR-NK cells exhibit great therapeutic potential in lymphohematopoietic malignancies, including DLBCL (67, 68). Our results demonstrated that DLBCL patients with high CD244 expression consistently exhibited lower TIDE scores across six independent cohorts. This may reflect a “hot tumor” phenotype in which abundant immune infiltration coexists with multiple co−expressed immune checkpoints, creating a highly immunosuppressive but potentially ICB−sensitive state (69). However, these are computational predictions and require validation in an ICB−treated DLBCL cohort. According to our study, CD244 may serve as a novel biomarker both for prognosis evaluation and treatment decisions in DLBCL.

**Figure 8:**
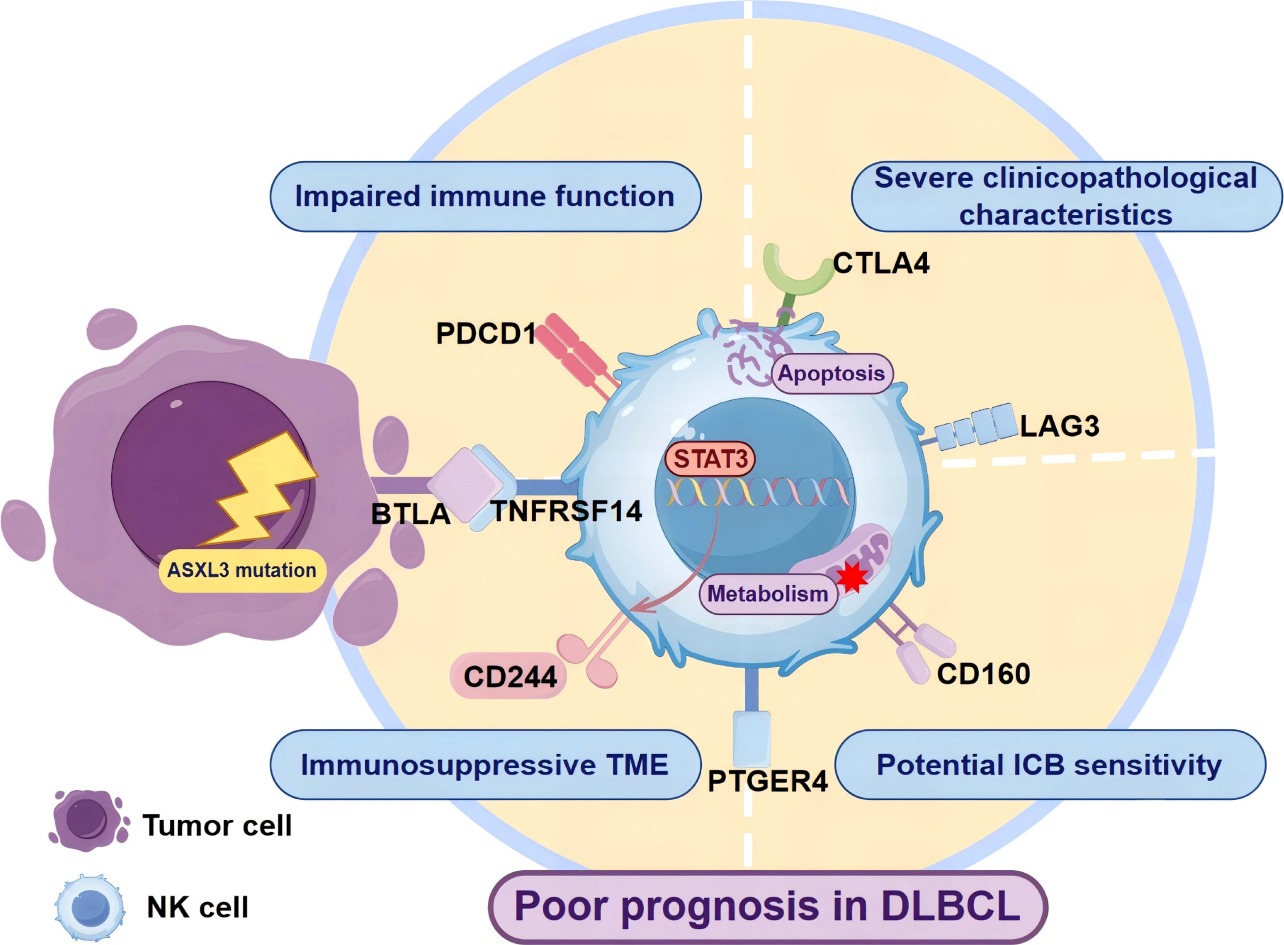
The predicted model of CD244-associated NK cell dysfunction and its significance in DLBCL (by Figdraw).

In conclusion, our study elucidated the expression pattern of CD244 in DLBCL, and NK cells were the main clusters that expressed CD244, accompanied by overexpression of canonical IC. EAT2 was identified as the main intracellular adaptor of CD244, and the related inhibitor signaling may be associated with metabolic alterations and immune deficiency in NK cells. High CD244 indicated immunosuppressive TME, severe clinicopathological features, adverse clinical outcomes, and increased susceptibility to ICB therapy. Furthermore, the CD244 expression may be regulated by STAT3 in NK cells and ASXL3 mutation in tumor cells through BTLA-TNFRSF14 pathway (**Figure 8**). Above all, our study clarified the significance of CD244 in NK cell dysfunction and DLBCL progression, which offered a feasible option for optimizing immunotherapy. However, further analysis on a larger sample size and functional studies on the role of CD244 in NK cell dysfunction are required to strengthen these findings.

## Data Availability

The original contributions presented in the study are included in the article/**Supplementary Material**. Further inquiries can be directed to the corresponding author.

## Supplementary material

Supplementary Figure 1 UMAP visualization based on sample origin **(a)** and clustering of 47 distinct cell populations identified by scRNA-seq **(b)**, including naive T cells (n = 4; Naive T-1, Naive T-2, Naive T-3, Naive T-4), CD4^+^TILs (n = 8; CD4–1 to CD4–8), CD8^+^TILs (n = 7; CD8–1 to CD8-7), B cells (n = 24; B-1 to B-24), macrophages, dendritic cells (DC-1, DC-2), and NK cells.

Supplementary Figure 2 Heatmap depicting the chromosomal CNV landscape **(a)** and comparison of total CNV burden across all B cell clusters in DLBCL samples versus controls **(b)**. The B-2 cluster from healthy donors was used as the reference for normal CNV levels.

Supplementary Table 1 The correlation between CD244 expression and TFs in NK cells from patients with DLBCL, as determined by scRNA-seq.

Supplementary Table 2 The correlation between TNFRSF14 expression and TFs in NK cells from patients with DLBCL, as determined by scRNA-seq.

## References

[1] Zhang J Wang D Luan M Liu X Wang N Sheng X . Liquid-liquid phase separation of ZHX2 protects DLBCL cells against ferroptosis through induction of SLC3A2. Leukemia. (2025) 39:2442–51. doi: 10.1038/s41375-025-02718-z 40730912 PMC12463659

[2] Dai Y Kizhakeyil A Chihara D Li X Liu Y Sainz Zuniga TP . Multi-modal spatial characterization of tumor immune microenvironments identifies targetable inflammatory niches in diffuse large B cell lymphoma. Nat Genet. (2025) 57:2715–27. doi: 10.1038/s41588-025-02353-5 41120574 PMC12597830

[3] Zhu Q Yang Y Chen K Zhang Q Huang Y Jian S . Diffuse large B-cell lymphoma: the significance of CD8(+) tumor-infiltrating lymphocytes exhaustion mediated by TIM3/Galectin-9 pathway. J Transl Med. (2024) 22:174. doi: 10.1186/s12967-024-05002-3 38369502 PMC10874540

[4] Tumuluru S Godfrey JK Cooper A Yu J Chen X MacNabb BW . Integrative genomic analysis of DLBCL identifies immune environments associated with bispecific antibody response. Blood. (2025) 145:2460–72. doi: 10.1182/blood.2024025355 39869833 PMC12163739

[5] Lue JK Healton SE Salles GA . Leveraging immunologically based therapies to treat diffuse large B-cell lymphoma. Trends Cancer. (2025) 11:1118–29. doi: 10.1016/j.trecan.2025.06.013 40707282 PMC12326298

[6] Wang Y Ding Q Wei J . Current advances and future directions of combined ICIs and TILs in solid tumors. Cancer Lett. (2026) 636:218145. doi: 10.1016/j.canlet.2025.218145 41241304

[7] Xu Y Zhang Q Gao J Yao S Tian C He T . Effects of immune checkpoint inhibitors on the pulmonary circulation in lung cancer patients. Int J Cancer. (2026) 158:682–96. doi: 10.1002/ijc.70110 40913348 PMC12670334

[8] Hanna A Sun X Sheng Q Gonzalez-Ericsson PI Wescott EC Taylor BC . Monitoring systemic immune responses to checkpoint inhibition in breast cancer reveals host responses and mechanisms of resistance. J Immunother Cancer. (2025) 13:e012606. doi: 10.1136/jitc-2025-012606 41371907 PMC12699608

[9] Pantelis P Tremoulis DC Evangelou K Bakouros P Magkouta S Ntintas OA . Immune cell senescence drives responsiveness to immunotherapy in melanoma. Mol Cancer. (2025) 24(1):308. doi: 10.1186/s12943-025-02517-1 41372921 PMC12717699

[10] Azlan A Zakaria N Rafsanjani MR Solayappan M Yik MY Said MSM . Current progress in the elucidation of Acute Myeloid Leukemia (AML) immune landscapes. Crit Rev Oncology/Hematology. (2025) 217:105002. doi: 10.1016/j.critrevonc.2025.105002 41197885

[11] Perales MA Awan FT Boumendil A Patel J Castagna L Angelucci E . Outcomes of allogeneic HCT in Hodgkin lymphoma in the era of checkpoint inhibitors: a joint CIBMTR and EBMT analysis. Blood. (2025) 146:1011–29. doi: 10.1182/blood.2024027197 40623049 PMC12530899

[12] Cristaldi V Lund AW . Structural vulnerabilities in DLBCL for enhanced treatment strategies. Cancer Res. (2023) 83:2643–4. doi: 10.1158/0008-5472.Can-23-1956 37404051

[13] Wu J Cheng J Zhu L Gao Q Lin H Zeng Y . CD5 ablation enhances persistence and antitumor potency of engineered T cells by mitigating exhaustion and promoting cytotoxicity. J Immunother Cancer. (2025) 13:e012243. doi: 10.1136/jitc-2025-012243 41320226 PMC12666075

[14] Cheng J Ge T Zhu X Wang J Zeng Y Mu W . Preclinical development and evaluation of nanobody-based CD70-specific CAR T cells for the treatment of acute myeloid leukemia. Cancer Immunology Immunotherapy CII. (2023) 72:2331–46. doi: 10.1007/s00262-023-03422-6 36932256 PMC10264288

[15] Agresta L Lehn M Lampe K Cantrell R Hennies C Szabo S . CD244 represents a new therapeutic target in head and neck squamous cell carcinoma. J Immunother Cancer. (2020) 8:e000245. doi: 10.1136/jitc-2019-000245 32217758 PMC7174077

[16] Georgoudaki AM Khodabandeh S Puiac S Persson CM Larsson MK Lind M . CD244 is expressed on dendritic cells and regulates their functions. Immunol Cell Biol. (2015) 93:581–90. doi: 10.1038/icb.2014.124 25643613

[17] Sun L Gang X Li Z Zhao X Zhou T Zhang S . Advances in understanding the roles of CD244 (SLAMF4) in immune regulation and associated diseases. Front Immunol. (2021) 12:648182. doi: 10.3389/fimmu.2021.648182 33841431 PMC8024546

[18] Wu Y Kuang DM Pan WD Wan YL Lao XM Wang D . Monocyte/macrophage-elicited natural killer cell dysfunction in hepatocellular carcinoma is mediated by CD48/2B4 interactions. Hepatol (Baltimore Md). (2013) 57:1107–16. doi: 10.1002/hep.26192 23225218

[19] Li D Xiong W Wang Y Feng J He Y Du J . SLAMF3 and SLAMF4 are immune checkpoints that constrain macrophage phagocytosis of hematopoietic tumors. Sci Immunol. (2022) 7:eabj5501. doi: 10.1126/sciimmunol.abj5501 35061505

[20] Farhangnia P Ghomi SM Mollazadehghomi S Nickho H Akbarpour M Delbandi AA . SLAM-family receptors come of age as a potential molecular target in cancer immunotherapy. Front Immunol. (2023) 14:1174138. doi: 10.3389/fimmu.2023.1174138 37251372 PMC10213746

[21] Steen CB Luca BA Esfahani MS Azizi A Sworder BJ Nabet BY . The landscape of tumor cell states and ecosystems in diffuse large B cell lymphoma. Cancer Cell. (2021) 39:1422–1437.e1410. doi: 10.1016/j.ccell.2021.08.011 34597589 PMC9205168

[22] Roider T Seufert J Uvarovskii A Frauhammer F Bordas M Abedpour N . Dissecting intratumour heterogeneity of nodal B-cell lymphomas at the transcriptional, genetic and drug-response levels. Nat Cell Biol. (2020) 22:896–906. doi: 10.1038/s41556-020-0532-x 32541878

[23] Zhu Q Yang Y Zeng Y Chen K Zhang Q Wang L . The significance of CD8(+) tumor-infiltrating lymphocytes exhaustion heterogeneity and its underlying mechanism in diffuse large B-cell lymphoma. Int Immunopharmacol. (2024) 137:112447. doi: 10.1016/j.intimp.2024.112447 38909497

[24] Yang Y Shu Y Qin Z Zeng Y Chen K Liu X . High oxidative phosphorylation represented by UQCRFS1 marks CD8 + tumor-infiltrating lymphocytes exhaustion in diffuse large B-cell lymphoma. Biol Direct. (2025) 20:92. doi: 10.1186/s13062-025-00684-1 40804681 PMC12345122

[25] Vaes RDW Reynders K Sprooten J Nevola KT Rouschop KMA Vooijs M . Identification of potential prognostic and predictive immunological biomarkers in patients with stage I and stage III non-small cell lung cancer (NSCLC): a prospective exploratory study. Cancers. (2021) 13:6259. doi: 10.3390/cancers13246259 34944879 PMC8699057

[26] Zou C Zhu C Guan G Guo Q Liu T Shen S . CD48 is a key molecule of immunomodulation affecting prognosis in glioma. OncoTargets Ther. (2019) 12:4181–93. doi: 10.2147/ott.S198762 31213836 PMC6549391

[27] Wei C Liu X Wang Q Li Q Xie M . Identification of hypoxia signature to assess the tumor immune microenvironment and predict prognosis in patients with ovarian cancer. Int J Endocrinol. (2021) 2021:4156187. doi: 10.1155/2021/4156187 34950205 PMC8692015

[28] Tan L Cheng D Wen J Huang K Zhang Q . Identification of prognostic hypoxia-related genes signature on the tumor microenvironment in esophageal cancer. Math Biosci Eng MBE. (2021) 18:7743–58. doi: 10.3934/mbe.2021384 34814273

[29] Hodgins JJ Khan ST Park MM Auer RC Ardolino M . Killers 2.0: NK cell therapies at the forefront of cancer control. J Clin Invest. (2019) 129:3499–510. doi: 10.1172/jci129338 31478911 PMC6715409

[30] Martinez-Perez A Aguilar-Garcia C Gonzalez S . The emerging role of NK cells in immune checkpoint blockade. Cancers. (2022) 14:6005. doi: 10.3390/cancers14236005 36497486 PMC9736655

[31] Worel N Grabmeier-Pfistershammer K Kratzer B Schlager M Tanzmann A Rottal A . The frequency of differentiated CD3(+)CD27(-)CD28(-) T cells predicts response to CART cell therapy in diffuse large B-cell lymphoma. Front Immunol. (2022) 13:1004703. doi: 10.3389/fimmu.2022.1004703 36700229 PMC9868136

[32] Klanova M Oestergaard MZ Trněný M Hiddemann W Marcus R Sehn LH . Prognostic impact of natural killer cell count in follicular lymphoma and diffuse large B-cell lymphoma patients treated with immunochemotherapy. Clin Cancer Res Off J Am Assoc For Cancer Res. (2019) 25:4634–43. doi: 10.1158/1078-0432.Ccr-18-3270 31053601

[33] Zare N Haghjooy Javanmard SH Mehrzad V Eskandari N Andalib AR . Effect of plasma-derived exosomes of refractory/relapsed or responsive patients with diffuse large B-cell lymphoma on natural killer cells functions. Cell J. (2020) 22:40–54. doi: 10.22074/cellj.2020.6550 31606965 PMC6791076

[34] Huo Z Chen F Zhao J Liu P Chao Z Liu K . Prognostic impact of absolute peripheral blood NK cell count after four cycles of R-CHOP-like regimen treatment in patients with diffuse large B cell lymphoma. Clin Exp Med. (2023) 23:4665–72. doi: 10.1007/s10238-023-01249-0 37938466 PMC10725372

[35] Beldi-Ferchiou A Jais JP Ghesquieres H Casasnovas RO Tilly H Fruchart C . Lenalidomide maintenance fails to overcome the unfavourable prognosis of low NK-cell counts in rituximab-chemotherapy responsive elderly DLBCL patients: a LYSA group study. Br J Haematol. (2023) 201:256–66. doi: 10.1111/bjh.18642 36740991

[36] Yang Z Yu W . Correction to: Clinical significance of circulating neutrophils and lymphocyte subsets in newly diagnosed patients with diffuse large B-cell lymphoma. Clin Exp Med. (2023) 23:823. doi: 10.1007/s10238-022-00881-6 35939174

[37] Vari F Arpon D Keane C Hertzberg MS Talaulikar D Jain S . Immune evasion via PD-1/PD-L1 on NK cells and monocyte/macrophages is more prominent in Hodgkin lymphoma than DLBCL. Blood. (2018) 131:1809–19. doi: 10.1182/blood-2017-07-796342 29449276 PMC5922274

[38] Azoulay T Slouzky I Karmona M Filatov M Hayun M Ofran Y . Compromised activity of natural killer cells in diffuse large b-cell lymphoma is related to lymphoma-induced modification of their surface receptor expression. Cancer Immunology Immunotherapy CII. (2023) 72:707–18. doi: 10.1007/s00262-022-03284-4 36048214 PMC10992952

[39] Agresta L Hoebe KHN Janssen EM . The emerging role of CD244 signaling in immune cells of the tumor microenvironment. Front Immunol. (2018) 9:2809. doi: 10.3389/fimmu.2018.02809 30546369 PMC6279924

[40] Kim J Kim TJ Chae S Ha H Park Y Park S . Targeted deletion of CD244 on monocytes promotes differentiation into anti-tumorigenic macrophages and potentiates PD-L1 blockade in melanoma. Mol Cancer. (2024) 23:45. doi: 10.1186/s12943-024-01936-w 38424542 PMC10903025

[41] Lutz V Hellmund VM Picard FSR Raifer H Ruckenbrod T Klein M . IL18 receptor signaling regulates tumor-reactive CD8+ T-cell exhaustion via activation of the IL2/STAT5/mTOR pathway in a pancreatic cancer model. Cancer Immunol Res. (2023) 11:421–34. doi: 10.1158/2326-6066.Cir-22-0398 36758176

[42] McNerney ME Lee KM Kumar V . 2B4 (CD244) is a non-MHC binding receptor with multiple functions on natural killer cells and CD8+ T cells. Mol Immunol. (2005) 42:489–94. doi: 10.1016/j.molimm.2004.07.032 15607804

[43] Vaidya SV Mathew PA . Of mice and men: different functions of the murine and human 2B4 (CD244) receptor on NK cells. Immunol Lett. (2006) 105:180–4. doi: 10.1016/j.imlet.2006.02.006 16621032

[44] Veillette A . NK cell regulation by SLAM family receptors and SAP-related adapters. Immunol Rev. (2006) 214:22–34. doi: 10.1111/j.1600-065X.2006.00453.x 17100873

[45] Buller CW Mathew PA Mathew SO . Roles of NK cell receptors 2B4 (CD244), CS1 (CD319), and LLT1 (CLEC2D) in cancer. Cancers. (2020) 12:1755. doi: 10.3390/cancers12071755 32630303 PMC7409338

[46] Bell HN Huber AK Singhal R Korimerla N Rebernick RJ Kumar R . Microenvironmental ammonia enhances T cell exhaustion in colorectal cancer. Cell Metab. (2023) 35:134–149.e136. doi: 10.1016/j.cmet.2022.11.013 36528023 PMC9841369

[47] Møller SH Hsueh PC Yu YR Zhang L Ho PC . Metabolic programs tailor T cell immunity in viral infection, cancer, and aging. Cell Metab. (2022) 34:378–95. doi: 10.1016/j.cmet.2022.02.003 35235773

[48] Dai L Fan G Xie T Li L Tang L Chen H . Single-cell and spatial transcriptomics reveal a high glycolysis B cell and tumor-associated macrophages cluster correlated with poor prognosis and exhausted immune microenvironment in diffuse large B-cell lymphoma. biomark Res. (2024) 12:58. doi: 10.1186/s40364-024-00605-w 38840205 PMC11155084

[49] Liu Y Kimpara S Hoang NM Daenthanasanmak A Li Y Lu L . EGR1-mediated metabolic reprogramming to oxidative phosphorylation contributes to ibrutinib resistance in B-cell lymphoma. Blood. (2023) 142:1879–94. doi: 10.1182/blood.2023020142 37738652 PMC10731920

[50] Yan ZX Dong Y Qiao N Zhang YL Wu W Zhu Y . Cholesterol efflux from C1QB-expressing macrophages is associated with resistance to chimeric antigen receptor T cell therapy in primary refractory diffuse large B cell lymphoma. Nat Commun. (2024) 15:5183. doi: 10.1038/s41467-024-49495-4 38890370 PMC11189439

[51] Che N Li M Liu X Cui CA Gong J Xuan Y . Macelignan prevents colorectal cancer metastasis by inhibiting M2 macrophage polarization. Phytomedicine. (2024) 122:155144. doi: 10.1016/j.phymed.2023.155144 37925889

[52] Jeong H Kim S Hong BJ Lee CJ Kim YE Bok S . Tumor-associated macrophages enhance tumor hypoxia and aerobic glycolysis. Cancer Res. (2019) 79:795–806. doi: 10.1158/0008-5472.Can-18-2545 30610087

[53] Le K Sun J Khawaja H Shibata M Maggirwar SB Smith MR . Mantle cell lymphoma polarizes tumor-associated macrophages into M2-like macrophages, which in turn promote tumorigenesis. Blood Adv. (2021) 5:2863–78. doi: 10.1182/bloodadvances.2020003871 34297045 PMC8341355

[54] Wang Y Zhang J Shi H Wang M Yu D Fu M . M2 tumor-associated macrophages-derived exosomal MALAT1 promotes glycolysis and gastric cancer progression. Advanced Sci (Weinheim Baden-Württemberg Germany). (2024) 11:e2309298. doi: 10.1002/advs.202309298 38639382 PMC11199979

[55] Wculek SK Cueto FJ Mujal AM Melero I Krummel MF Sancho D . Dendritic cells in cancer immunology and immunotherapy. Nat Rev Immunol. (2020) 20:7–24. doi: 10.1038/s41577-019-0210-z 31467405

[56] Cooper L Xu H Polmear J Kealy L Szeto C Pang ES . Type I interferons induce an epigenetically distinct memory B cell subset in chronic viral infection. Immunity. (2024) 57:1037–1055.e1036. doi: 10.1016/j.immuni.2024.03.016 38593796 PMC11096045

[57] Wenzl K Stokes ME Novak JP Bock AM Khan S Hopper MA . Multiomic analysis identifies a high-risk signature that predicts early clinical failure in DLBCL. Blood Cancer J. (2024) 14:100. doi: 10.1038/s41408-024-01080-0 38902256 PMC11189905

[58] Sordo-Bahamonde C Lorenzo-Herrero S Granda-Díaz R Martínez-Pérez A Aguilar-García C Rodrigo JP . Beyond the anti-PD-1/PD-L1 era: promising role of the BTLA/HVEM axis as a future target for cancer immunotherapy. Mol Cancer. (2023) 22:142. doi: 10.1186/s12943-023-01845-4 37649037 PMC10466776

[59] Wojciechowicz K Spodzieja M Wardowska A . The BTLA-HVEM complex - the future of cancer immunotherapy. Eur J Med Chem. (2024) 268:116231. doi: 10.1016/j.ejmech.2024.116231 38387336

[60] Guruprasad P Carturan A Zhang Y Cho JH Kumashie KG Patel RP . The BTLA-HVEM axis restricts CAR T cell efficacy in cancer. Nat Immunol. (2024) 25:1020–32. doi: 10.1038/s41590-024-01847-4 38831106

[61] Sordo-Bahamonde C Lorenzo-Herrero S Gonzalez-Rodriguez AP Á R P González-García E López-Soto A . BTLA/HVEM axis induces NK cell immunosuppression and poor outcome in chronic lymphocytic leukemia. Cancers. (2021) 13:1766. doi: 10.3390/cancers13081766 33917094 PMC8067870

[62] Sun H Kim E Ryu J Lee H Shin EA Lee M . TM4SF5-mediated liver Malignancy involves NK cell exhaustion-like phenotypes. Cell Mol Life Sciences: CMLS. (2021) 79:49. doi: 10.1007/s00018-021-04051-x 34921636 PMC8739317

[63] Cianciotti BC Magnani ZI Ugolini A Camisa B Merelli I Vavassori V . TIM-3, LAG-3, or 2B4 gene disruptions increase the anti-tumor response of engineered T cells. Front Immunol. (2024) 15:1315283. doi: 10.3389/fimmu.2024.1315283 38510235 PMC10953820

[64] Khan M Arooj S Wang H . NK cell-based immune checkpoint inhibition. Front Immunol. (2020) 11:167. doi: 10.3389/fimmu.2020.00167 32117298 PMC7031489

[65] Cai L Li Y Tan J Xu L Li Y . Targeting LAG-3, TIM-3, and TIGIT for cancer immunotherapy. J Hematol Oncol. (2023) 16:101. doi: 10.1186/s13045-023-01499-1 37670328 PMC10478462

[66] Wang F Liu S Liu F Xu T Ma J Liang J . TIGIT immune checkpoint blockade enhances immunity of human peripheral blood NK cells against castration-resistant prostate cancer. Cancer Lett. (2023) 568:216300. doi: 10.1016/j.canlet.2023.216300 37414394

[67] Liu E Marin D Banerjee P Macapinlac HA Thompson P Basar R . Use of CAR-transduced natural killer cells in CD19-positive lymphoid tumors. N Engl J Med. (2020) 382:545–53. doi: 10.1056/NEJMoa1910607 32023374 PMC7101242

[68] Duell J Westin J . The future of immunotherapy for diffuse large B-cell lymphoma. Int J Cancer. (2025) 156:251–61. doi: 10.1002/ijc.35156 39319495 PMC11578085

[69] Jiang P Gu S Pan D Fu J Sahu A Hu X . Signatures of T cell dysfunction and exclusion predict cancer immunotherapy response. Nat Med. (2018) 24:1550–8. doi: 10.1038/s41591-018-0136-1 30127393 PMC6487502

