# Supplementary figures and images for: CD244 overexpression indicates NK cell dysfunction and tumor progression in diffuse large B-cell lymphoma
Source: Front Immunol. 2026 Jul 7;17:1855521. doi: 10.3389/fimmu.2026.1855521 (PMC13384930; doi:10.3389/fimmu.2026.1855521)

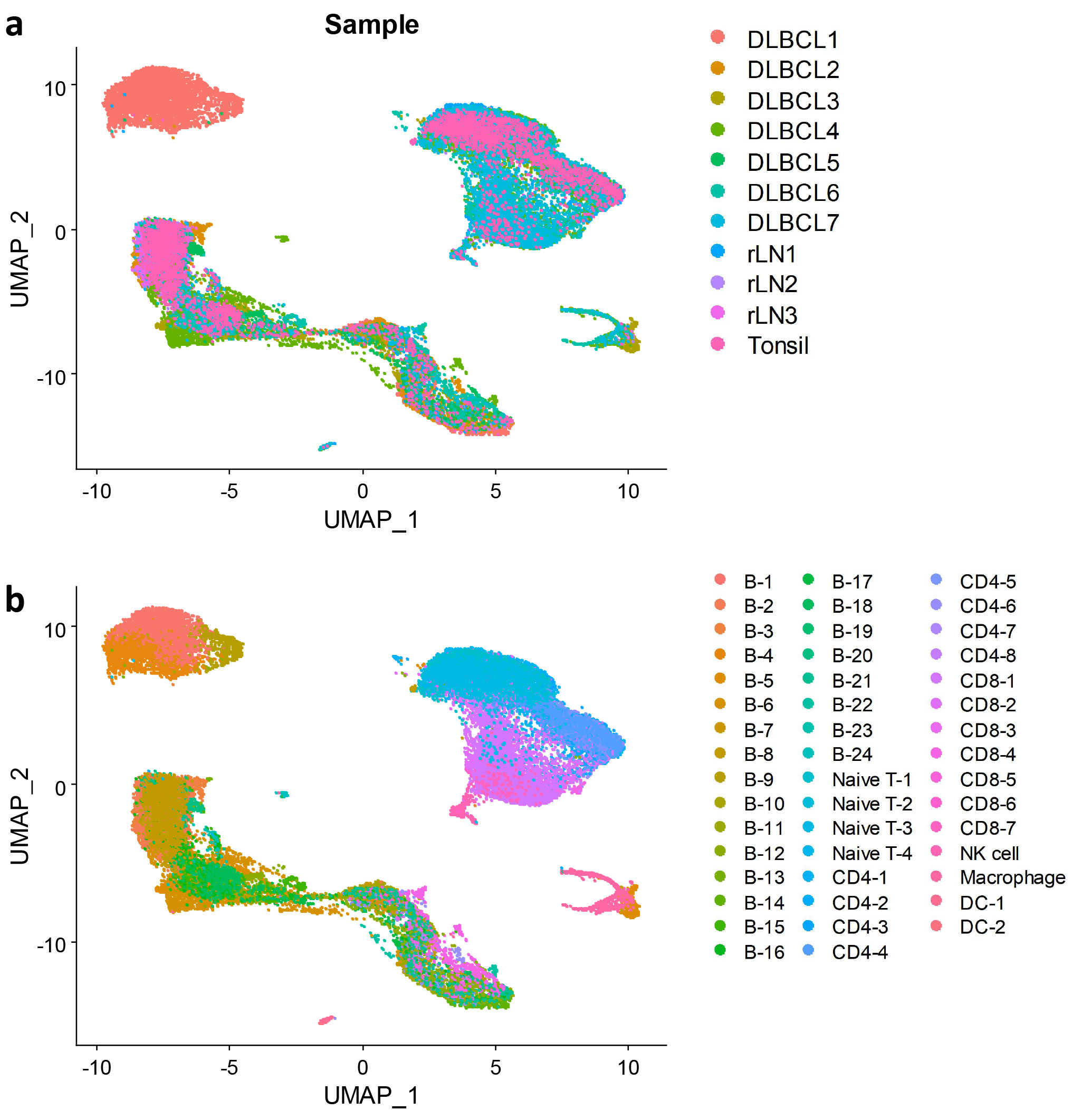

Supplement: Supplementary Figure 1 — UMAP visualization based on sample origin (a) and clustering of 47 distinct cell populations identified by scRNA-seq (b), including naive T cells (n = 4; Naive T-1, Naive T-2, Naive T-3, Naive T-4), CD4+TILs (n = 8; CD4–1 to CD4–8), CD8+TILs (n = 7; CD8–1 to CD8-7), B cells (n = 24; B-1 to B-24), macrophages, dendritic cells (DC-1, DC-2), and NK cells. [file Image1.jpeg]

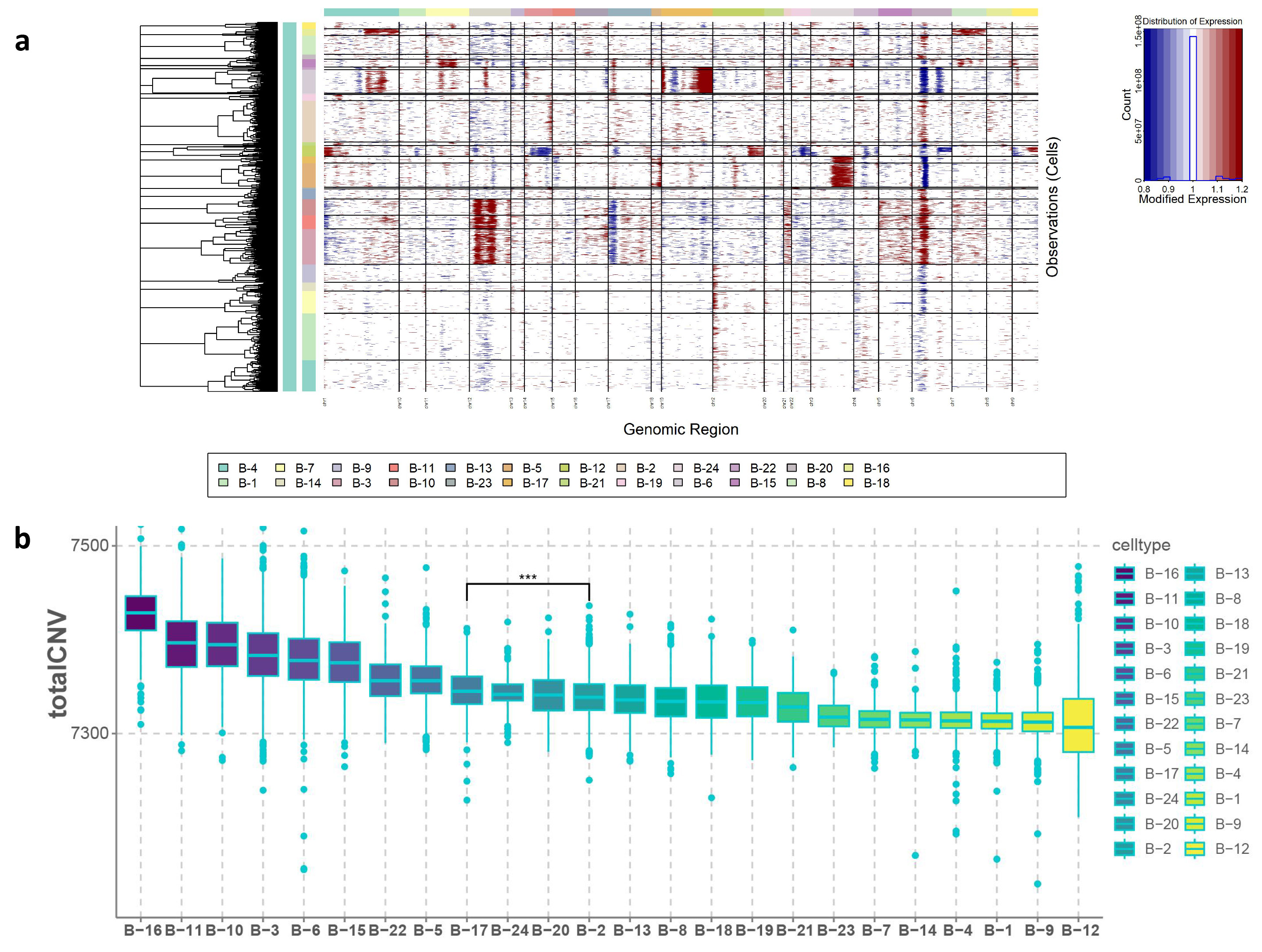

Supplement: Supplementary Figure 2 — Heatmap depicting the chromosomal CNV landscape (a) and comparison of total CNV burden across all B cell clusters in DLBCL samples versus controls (b). The B-2 cluster from healthy donors was used as the reference for normal CNV levels. [file Image2.jpeg]
