# Supplementary material for: CD244 overexpression indicates NK cell dysfunction and tumor progression in diffuse large B-cell lymphoma
Source: Front Immunol. 2026 Jul 7;17:1855521. doi: 10.3389/fimmu.2026.1855521 (PMC13384930; doi:10.3389/fimmu.2026.1855521)
Supplement: Supplementary Table 1 — The correlation between CD244 expression and TFs in NK cells from patients with DLBCL, as determined by scRNA-seq. [file Table1.docx]

Supplementary Table 1. The correlation between CD244 expression and TFs in NK cells from patients with DLBCL, as determined by scRNA-seq.

| **Transcription factors** | **correlation** | **p value** |
| --- | --- | --- |
| TOX | 0.250110104 | 2.30E-05 |
| LITAF | 0.231004911 | 9.58E-05 |
| STAT3 | 0.157620489 | 0.008236138 |
| STAT5A | 0.148027142 | 0.013154379 |
| EZH2 | 0.145106873 | 0.015094 |
| RORA | 0.135397797 | 0.023454594 |
| BATF | 0.13128677 | 0.028053591 |
| TBX21 | 0.130188191 | 0.029406238 |
| TCF7 | -0.120622831 | 0.043722012 |
