# Supplementary material for: CD244 overexpression indicates NK cell dysfunction and tumor progression in diffuse large B-cell lymphoma
Source: Front Immunol. 2026 Jul 7;17:1855521. doi: 10.3389/fimmu.2026.1855521 (PMC13384930; doi:10.3389/fimmu.2026.1855521)
Supplement: Supplementary Table 2 — The correlation between TNFRSF14 expression and TFs in NK cells from patients with DLBCL, as determined by scRNA-seq. [file Table2.docx]

Supplementary Table 2. The correlation between TNFRSF14 expression and TFs in NK cells from patients with DLBCL, as determined by scRNA-seq.

| **Transcription factors** | **correlation** | **p value** |
| --- | --- | --- |
| TOX | 0.237996827 | 5.75632E-05 |
| STAT3 | 0.187706243 | 0.001605173 |
| ZMIZ2 | 0.154558207 | 0.009590152 |
| NR4A3 | -0.140395036 | 0.018753158 |
| ATF1 | 0.133721091 | 0.025245149 |
| SP100 | 0.128746147 | 0.031266178 |
| EOMES | 0.123299094 | 0.03922368 |
| STAT5B | 0.118349906 | 0.047875471 |
